# Safety and efficacy of umbilical cord-derived Wharton’s jelly compared to hyaluronic acid and saline for knee osteoarthritis: study protocol for a randomized, controlled, single-blind, multi-center trial

**DOI:** 10.1186/s13018-021-02475-6

**Published:** 2021-05-31

**Authors:** Ashim Gupta, Nicola Maffulli, Hugo C. Rodriguez, Eric W. Carson, Randa A. Bascharon, Kristin Delfino, Howard J. Levy, Saadiq F. El-Amin

**Affiliations:** 1 BioIntegrate, Lawrenceville, GA USA; 2 Future Biologics, Lawrenceville, GA USA; 3 South Texas Orthopaedic Research Institute, Laredo, TX USA; 4 Veterans in Pain, Los Angeles, CA USA; 5 Department of Musculoskeletal Disorders, School of Medicine and Surgery, University of Salerno, Fisciano, Italy; 6 San Giovanni di Dio e Ruggi D’Aragona Hospital “Clinica Orthopedica” Department, Hospital of Salerno, Salerno, Italy; 7 Barts and the London School of Medicine and Dentistry, Centre for Sports and Exercise Medicine, Queen Mary University of London, London, UK; 8 School of Pharmacy and Bioengineering, Keele University School of Medicine, Stoke-on-Trent, UK; 9 Future Physicians of South Texas, San Antonio, TX USA; 10 University of the Incarnate Word, School of Osteopathic Medicine, San Antonio, TX USA; 11 Department of Orthopaedic Surgery, Washington University School of Medicine, St. Louis, MO USA; 12 Orthopedic & Sports Medicine Institute of Las Vegas, Las Vegas, NV USA; 13 Southern Illinois University, School of Medicine, Springfield, IL USA; 14 Department of Orthopaedic Surgery, Lenox Hill Hospital, Northwell Health, New York, NY USA; 15 El-Amin Orthopaedic and Sports Medicine Institute, 2505 Newpoint Pkwy, Suite – 100, Lawrenceville, GA 30043 USA

**Keywords:** Umbilical cord, Wharton’s jelly, Knee osteoarthritis, Regenerative medicine, Biologics, Randomized controlled trial, Extracellular vesicles, Exosomes, Growth factors, Hyaluronic acid

## Abstract

**Background:**

Osteoarthritis (OA) is the most common joint disorder in the United States of America (USA) with a fast-rising prevalence. Current treatment modalities are limited, and total knee replacement surgeries have shown disadvantages, especially for grade II/III OA. The interest in the use of biologics, including umbilical cord (UC)-derived Wharton’s jelly (WJ), has grown in recent years. The results from a preliminary study demonstrated the presence of essential components of regenerative medicine, namely growth factors, cytokines, hyaluronic acid (HA), and extracellular vesicles, including exosomes, in WJ. The proposed study aims to evaluate the safety and efficacy of intra-articular injection of UC-derived WJ for the treatment of knee OA symptoms.

**Methods:**

A randomized, controlled, single-blind, multi-center, prospective study will be conducted in which the safety and efficacy of intra-articular administration of UC-derived WJ are compared to HA (control) and saline (placebo control) in patients suffering from grade II/III knee OA. A total of 168 participants with grade II or III knee OA on the KL scale will be recruited across 53 sites in the USA with 56 participants in each arm and followed for 1 year post-injection. Patient satisfaction, Numeric Pain Rating Scale, Knee Injury and Osteoarthritis Outcome Score, 36-Item Short Form Survey (SF-36), and 7-point Likert Scale will be used to assess the participants. Physical exams, X-rays, and MRI with Magnetic Resonance Observation of Cartilage Repair Tissue score will be used to assess improvement in associated anatomy.

**Discussion:**

The study results will provide valuable information into the safety and efficacy of intra-articular administration of Wharton’s jelly for grade II/III knee osteoarthritis. The results of this study will also add to the treatment options available for grade II/III OA as well as help facilitate the development of a more focused treatment strategy for patients.

**Trial registration:**

ClinicalTrials.gov, NCT04711304. Registered on January 15, 2021

## Background

Osteoarthritis (OA) is the most common joint disorder in the United States of America (USA), affecting approximately 12% of US adults aged between 25 and 74 years [1]. By 2030, the number of US adults with arthritis is expected to reach 67 million, leading to a continuous increase in the number of total knee replacement surgeries [2–4]. While total knee replacement surgeries have shown advantages, avoiding or delaying such surgery is usually desirable, for medical reasons and health care system perspective [5]. The long-term outcomes after total knee replacement surgeries for patients with grade II or III knee OA on the Kellgren-Lawrence (KL) scale are worse compared to patients with grade IV OA [6, 7]. Additionally, conventional treatment modalities, including activity modification, physical therapy, and pharmacological agents such as non-steroidal anti-inflammatory drugs, corticosteroids, viscosupplementation, and narcotics, have limitations and potential side effects [8–16]. Thus, there is a need for alternative intervention for patients with grade II or III knee OA.

Interest in the use of biologics for regenerative medicine applications has increased over the last decade [17–22]. To be compliant with the relevant laws and regulations in the US biologics that adhere to the US Food and Drug Administration regulation of Human Cells, Tissues, and Cellular and Tissue-Based Products (HCT/P’s) regulated under title 21, part 1271 of the Code of Federal Regulations, must meet all the conditions under section 361 of Public Health Safety Act to be regulated solely under this section [17, 23]. According to this regulation, HCT/P’s must meet the criteria of being minimally manipulated, for homologous use only, not to be combination products, to have no systemic effect, and to be non-dependent on the metabolic activity of the living cells [17]. Despite the increased use, there is insufficient literature assessing the amount of growth factors (GFs), cytokines (CKs), hyaluronic acid (HA), and extracellular vesicles (EVs), including exosomes, present in these products, and more specifically, umbilical cord (UC)-derived Wharton’s jelly (WJ). In addition, there is limited or no literature assessing the safety and efficacy of UC-derived WJ products via a randomized, controlled, multi-center study.

We formulated a novel UC-derived WJ product that has been shown to contain the essential components of regenerative medicine, namely GFs, CKs, HA, and EVs [24]. In addition, the Wharton’s jelly has been reported to contain high amounts of extracellular matrix components, including collagen, hyaluronic acid, and sulfated proteoglycans, required to improve the treatment effect [24]. That study was an essential preliminary step to better characterize the WJ formulation before performing clinical trials to determine its safety and efficacy including providing symptomatic relief to patients with grade II or III knee OA.

The goal of the proposed study is to evaluate the safety and efficacy of intra-articular injection of UC-derived WJ for the treatment of knee OA symptoms. We hypothesize that there will be no difference in the outcomes in patients receiving injection of UC-derived WJ, HA, or saline in terms of safety. We also hypothesize that patients receiving intra-articular injection of WJ will show an improvement in their overall satisfaction, Numeric Pain Rating Scale (NPRS), Knee Injury and Osteoarthritis Outcome Score (KOOS), and cartilage formation over a period of 1 year compared to the baseline visit. Our null hypothesis is that there is no difference in patients receiving either WJ, HA, or saline, and no difference between baseline and after treatment within each treatment group over a period of 1 year.

## Methods and analysis

This study protocol is reported in accordance with the Standard Protocol Items: Recommendations for Intervention Trials (SPIRIT) criteria [25, 26].

### Study setting

This multi-center study involves up to 53 sites consisting of health care centers, community clinics, and academic hospitals in the USA.

### Study design

**Fig. 1:**
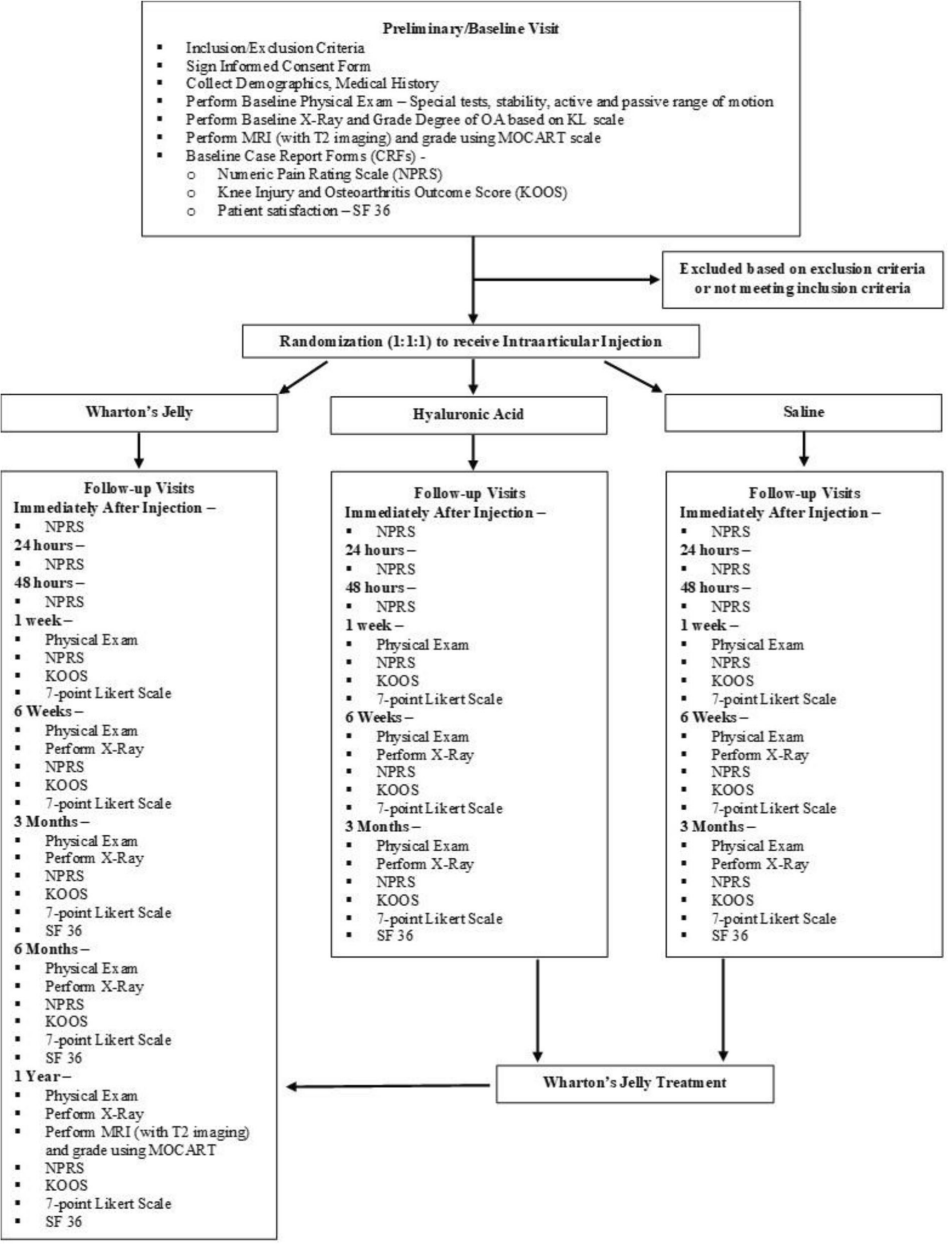
Summary of the trial design

This is a randomized, controlled, single-blind, multi-center, prospective study in which the safety and efficacy of intra-articular UC-derived WJ are compared to HA (control) and saline (placebo control) in patients suffering with knee OA. The participants in the treatment arm will receive a 2-mL intra-articular injection of UC-derived WJ (GeneXSTEM, BioIntegrate Inc., Lawrenceville, GA, USA, diluted with 1:1 sterile normal saline). The participants in the control arm and placebo arm will receive 4 mL HA (Monovisc®—4 mL injection with 22mg/mL HA, Anika Therapeutics, Bedford, MA, USA) and 4 mL of sterile normal saline, respectively. Patients in the HA group or saline group will be offered the opportunity to cross over to the Wharton’s jelly group after 3 months, as a method to aid in study recruitment and retention (Fig. 1).

### Participants

A total of 168 patients with grade II or III knee OA on the KL scale will be recruited with 56 patients in each arm. Participation will be discussed with patients who meet the inclusion criteria. The patients will be given the opportunity to read an informed consent form (ICF) and obtain answers to all questions before considering participation at the enrollment/baseline visit.

#### Inclusion criteria

Consenting adult patients over the age of 18 years diagnosed with grade II or grade III (mild or moderate) OA on the KL scale (in only one knee) will be considered for this study along with the following inclusion criteria:

- Body mass index (BMI) of <50kg/m^2^.
- Ability to comply with requirements of study visits.
- Pain score of 4 or more on the Numeric Pain Rating Scale (NPRS).
- Female patients must be abstinent, surgically sterilized, or postmenopausal.
- Premenopausal females must have a negative pregnancy test, on contraceptive measures, and do not anticipate pregnancy during the duration of the study.
- Males with premenopausal female partners will have to take contraceptive measures for the duration of the study.
- Be willing and capable of giving written informed consent to participate.
- Be willing and capable of complying with study-related requirements, procedures, and visits.

#### Exclusion criteria

Patients will be excluded from the enrollment in the study if they meet any of the following exclusion criteria:

- Patients who have taken any pain medication including non-steroidal anti-inflammatory drugs (NSAIDs) within 2 weeks prior to study injection date
- Patients who use anticoagulants, have a substance abuse history, and/or fail to agree not to take any knee symptom-modifying drugs during the course of the study without discussing and reporting the use to the site principal investigator and study team
- Positive on special tests and/or stability tests on physical exam
- Patients with intra-articular injection of any drug including corticosteroids and viscosupplementation in the index knee in the last 3 months
- Surgery on the index knee within the last 6 months
- Traumatic injury to the index knee within the last 3 months
- Planned elective surgery during the course of the study
- History of organ or hematologic transplantation, rheumatoid arthritis, or other autoimmune disorders
- Patients on immunosuppressive medications/treatment
- Patients with a diagnosis of non-basal cell carcinoma within the last 5 years
- Patients with knee infection or who used antibiotics for knee infection within the last 3 months
- Patients who participated in another clinical trial or treatment with any investigational product within the last 30 days prior to the inclusion in the study
- Female patients who are breastfeeding or are pregnant or desire to be pregnant or become pregnant during the course of the study
- Contraindications to radiographic or MRI imaging
- Serious neurological, psychological, or psychiatric disorders
- Other medical conditions including any malignancies determined by the site principal investigator as interfering with the study
- Injury or disability claims under current litigation or pending or approved workers’ compensation claims

Participants can voluntarily withdraw from the study at any time. Withdrawal from the study will not affect the patient’s access to other treatments nor will the patient be subjected to any sanctions. Participation in the study may be terminated if continued participation in the study is not in the subject’s best interest, according to the principal investigator’s opinion or if the subject withdraws participation. Determination of therapy cessation and need of explanation will be performed by the principal investigator (PI) based on the standard medical practice. Any patient who suffers an adverse event whether or not related to treatment may withdraw voluntarily.

### Randomization

Participants will be assigned to a treatment group using sealed opaque envelopes coded with an alphanumeric identifier to ensure consecutive allocation of envelopes. Block randomization across all sites will be used to ensure even distribution to each group of 1:1:1 allocation to the 3 study arms—WJ, HA, and saline.

### Study interventions

After completion of visit 1 (preliminary/baseline), and determination of patient’s eligibility to be enrolled in the study, participants will be randomized into one of the three arms of the study and be scheduled for the procedure visit. At this visit, the participants will either receive an intra-articular injection of WJ (treatment), HA (control), or saline (placebo control). Following the procedure, the participants will be periodically followed for 1 year. After the 3-month follow-up visit, patients injected with HA or saline will be offered the opportunity to cross over to the WJ group. If they decide to cross over, they will receive an injection of WJ and will follow the schedule of events for the WJ injection group beginning immediately after the injection follow-up visit.

### Assessment points

Assessments for the study period will begin at visit 1 (preliminary/baseline). The patients will be assessed using the inclusion/exclusion criteria for eligibility in the study. The participants will then undergo a physical exam (PE) on the knee diagnosed with grade II/III OA, as well as a baseline plain radiograph for an up-to-date OA grade on the KL scale and a T2-weighted MRI to obtain a Magnetic Resonance Observation of Cartilage Repair Tissue (MOCART) score. The participants will also be required to complete baseline case report forms (CRFs) including the Numeric Pain Rating Scale (NPRS), Knee Injury and Osteoarthritis Outcome Score (KOOS), and patient satisfaction/survey, SF-36. Demographic information and medical history will also be collected. The participants will then be assessed for any adverse events at visit 2.2, immediately following their respective intra-articular injection, and at their procedure visit (visit 2.1) and be re-assessed for pain using the NPRS. Through visit 3 (24 h follow-up), visit 4 (48 h follow-up), and visit 5 (1 week follow-up), patients will be reassessed for pain using the NPRS. On visit 5 (1 week follow-up), visit 6 (6 weeks follow-up), and visit 7 (3 months follow-up), participants will undergo another PE as well as have their NPRS, KOOS, and 7-point Likert scale recorded. Plain radiographs will be taken on visits 6 and 7. At this point of the study, patients in the HA or saline arms will be offered the opportunity to cross over to the WJ arm and will follow the schedule of events beginning at visit 2.2. On visit 8 (6 months follow-up) and visit 9 (1 year follow-up), patients will undergo a PE as well as have their NPRS, KOOS, 7 point Likert scale, and SF-36 taken and recorded. In addition, plain radiographs will be taken again followed by an MRI on visit 9 for a MOCART score. All participants will have the opportunity to report any adverse events at each visit or at any time within the study.

The following are the primary endpoints:

1. To determine the safety of umbilical cord-derived Wharton’s jelly formulation (GeneXSTEM™)
2. To assess the patient satisfaction

The following are the secondary endpoints:

- To assess the change in patient-reported outcome measures, NPRS and KOOS, from baseline and between the groups at different time points
- To assess the cartilage formation via MOCART at 1 year time point and compare it from baseline and between the groups

### Sample size and statistical analysis

A sample size calculation based on the initial analysis of change from baseline to 3 months was computed to determine the number needed per group to detect significance at the *α* = 0.05 level. To detect differences of 8–10 units in the KOOS, considered as the minimum clinical important difference (MCID), assuming an *α* of 0.05, *β* of 90%, and a 2-tailed test, the estimated sample size was calculated to be at least 40 subjects in each group, increased to 56 subjects in each group to allow for loss to follow-up. An interim analysis at the initial 3-month follow-up will allow us to examine the effect size and increase enrollment if necessary. The data will also be analyzed based on the age group, gender, and grade of osteoarthritis.

### Data collection and handling

All source documents will be maintained by the PI. Data will be transcribed on study CRFs, and the original data will be secured by the PI and made available to the sponsor and study monitors. The PI will maintain records for 5 years. All CRF pages will be subjected to the initial inspection for omitted data, data inconsistencies, illegible data, and deviations by the study monitors. All hard copies of CRFs and media will be stored in a secure location. The PI will be responsible for submitting the following data and reports:

- Adverse events (AEs): on an ongoing basis via the proper section of the CRF.
- Severe adverse events (SAEs) will be reported within 24 h of knowledge of the event to the sponsor and reported to IRB within 5 days, as per regulations.
- Any deviations, exceptions, and violations of protocol will be reported to the sponsor within 5 days and reported to IRB per their regulations.
- A protocol progress report will be provided to the sponsor and IRB as per regulations.
- A study closure report will be provided to the sponsor and IRB as per regulations.

### Quality control and assurance

All documents and data will be produced and maintained in such a way as to ensure control of documents and data to protect patients’ privacy as far as reasonably practicable. The sponsor, study monitor, and representatives of regulatory authorities are permitted to access the study documents (protocol, CRFs, medical records/files) as needed.

## Discussion

OA is a debilitating condition that affects millions of patients across the world, and it is estimated to drastically increase in prevalence in the upcoming years [1, 27]. OA can lead to marked pain, loss of independence, and significant health care costs [27, 28]. Currently, there are several non-operative treatment options available for grade II/III knee OA that aim to help reduce pain and enhance the quality of life, but unfortunately fail to resolve the underlying pathophysiological process of OA. These are the several reasons why the field of regenerative medicine and the use of biologics including UC-derived WJ has increased so profoundly.

The proposed clinical trial will be one of the first to examine the safety and efficacy of intra-articular administration of WJ compared to HA and saline in patients with grade II/III OA. Studies have demonstrated the efficacy of HA injections in controlling the signs and symptoms of OA, specifically in terms of pain and function, and reported safety and efficacy for the treatment of pain of OA of the knee in patients who have failed to adequately respond to conservative non-pharmacological therapy and simple analgesics such as acetaminophen [29, 30]. In addition, a recent study by Farr et al. also utilized HA as a control demonstrating the superiority of amniotic suspension allograft over HA and saline for modification of knee OA symptoms [5]. A recent meta-analysis also details the safety and efficacy of HA compared to corticosteroids for knee OA [31]. Thus, HA was chosen as a control for this proposed study.

On the other hand, despite the widespread use of HA, there is inconsistency in clinical studies pertaining to the effect of HA in knee OA. More randomized controlled trials with larger data set are required to test the efficacy of HA versus other established therapies of OA. Thus, both the Osteoarthritis Research Society International 2012 guideline and the American College of Rheumatology 2013 guidelines neither recommend nor discourage the use of HA [32]. Due to these reasons, this study is designed with an intent-to-treat, and therefore, the participants receiving HA or saline will be offered the opportunity to cross over to the Wharton’s jelly group after 3 months. Furthermore, the anecdotal evidence and unpublished case report from our team demonstrated that one injection is sufficient. The aforementioned study by Farr et al. also utilized one injection [5]. Therefore, in this study, we also proposed to utilize one injection only.

We anticipate that this study will demonstrate that intra-articular administration of WJ is safe. Some minimal adverse effects can be observed. The adverse events associated with the intra-articular administration of Wharton’s jelly will be similar to inherent risks associated with any intra-articular injection. These include pain and/or reaction at the injection site, failure of therapy to work as expected, infections, potential for contamination of the product, and unknown or unexpected reactions, including but not limited to immunogenic reactions, tumorigenic reactions, and development of autoimmune disorders. We also anticipate that patients suffering from grade II/III OA will experience improvement in their pain, function, quality of life, and overall satisfaction. In addition, we foresee that articular cartilage formation will increase over the 1-year period of the study in comparison with the baseline visit. This study has several limitations. The present investigation was designed as single-blinded rather than double-blinded. The concept of a double-blinded study was abandoned as the treating investigators can easily detect the difference in viscosity between the injectables used. Nevertheless, most of the endpoints in the study will be patient-reported, thus reducing the bias from the unblinded investigators. Another limitation of the study is the utilization of only one HA formulation: we are aware that the HA formulations with different molecular weights or cross-linking are available. Thus, the results from this study may not be applicable to other HA formulations. In conclusion, the results of this study will add to our understanding of the treatment options available for grade II/III OA and help facilitate the development of a more focused treatment strategy for patients with grade II/III OA.

## Data Availability

The datasets used and/or analyzed during the future study will be available from the corresponding author on reasonable request.

## Abbreviations

AEs: Adverse events
CKs: Cytokines
CRFs: Case report forms
EVs: Extracellular vesicles
GFs: Growth factors
HA: Hyaluronic acid
HCT/P’s: Human cells, tissues, and cellular and tissue-based products
KL: Kellgren-Lawrence
KOOS: Knee Injury and Osteoarthritis Outcome Score
SF-36: 36-Item Short Form Survey
MOCART: Magnetic Resonance Observation of Cartilage Repair Tissue
NPRS: Numeric Pain Rating Scale
OA: Osteoarthritis
SAEs: Severe adverse events
SPIRT: Standard Protocol Items-Recommendations for Intervention Trials
UC: Umbilical cord
WJ: Wharton’s jelly
